# Health Co-morbidities and Early Marriage in Women of a Rural Area of Nepal: A Descriptive Cross-Sectional Study

**DOI:** 10.31729/jnma.5205

**Published:** 2020-10-31

**Authors:** Naresh Manandhar, Sunil Kumar Joshi

**Affiliations:** 1 Department of Community Medicine, Kathmandu Medical College, Sinamangal

**Keywords:** *depression*, *early marriage*, *gynaecological*, *haemorrhage*, *miscarriage*

## Abstract

**Introduction:**

Early marriage is defined as the marriage of a young person less than 18 years. Early marriage is more prevalent in South Asia as more than half of all child marriage occurs here. Thirty-seven percent of girls in Nepal marry before age 18 years. This study was done to find out the health consequences of early marriage in women of a rural area of Nepal.

**Methods:**

A descriptive cross-sectional study was conducted from 10^th^ to 15^th^ Feb 2020 February in 358 women from Panauti, Kavrepalchowk. The convenient sampling method was used. Ethical approval was taken from the Institutional Review Committee. Economic status was assessed by using Kuppuswamy's socioeconomic scale. The collected data were analyzed using the Statistical Package for Social Science version 20. Point estimate at 95% confidence interval was calculated along with frequency and proportion for binary data.

**Results:**

The prevalence of early marriage was 187 (52.2%) (47.03 to 57.37 at 95% confidence interval). One hundred sixteen (62%) early marriage women had gynecological problems followed by depression problem 85 (45.5%) and miscarriage 32 (17.1%). The mean age of marriage was 17.2 years. The majority, i.e. 167 (89.3%) of respondents who married earlier were Hindu by religion. Early marriage was observed in 104 (55.6%) of illiterate women.

**Conclusions:**

The prevalence of early marriage was high. Early married women had a lower level of socio-economic status, lower level of education, which harmed the participants' health status.

## INTRODUCTION

Early marriage is defined as the marriage of a young person less than 18 years. Early marriage is more prevalent in South Asia as more than half of all child marriage occurs here.^1^ Nepal has the third-highest rate of child marriage in Asia, after Bangladesh and India.

Early marriage is more common in females than in males. Thirty-seven percent of girls in Nepal marry before age 18 and 10 percent are married by age 15, even though the minimum age of marriage under Nepali law is 20 years of age.^2^ Early marriage is not only a serious human rights violation driven by socio-cultural factors and poverty but also a significant barrier to women's health because girls have not yet attained full maturity and the capacity to act autonomously.^3^

This study was carried out to find out the prevalence of early marriage and its impact on women's health.

## METHODS

A descriptive cross-sectional study was conducted from 10^th^ to 15^th^ Feb 2020 February in Panauti, Kavrepalchowk. Ethical approval was taken from the Institutional Review Committee (Ref no: 1301202012) of Kathmandu Medical College. Married women age less than 50 years were included in the study. Unmarried women more than 50 years and women who did not give consent were excluded from the study.

Sample size calculation,

$$\begin{array}{ll} \text{n} & \text{= }{\text{Z}}^{\text{2}}\times \text{p}\times \text{(1}-\text{p)}/{\text{e}}^{\text{2}}\\ & \text{= }{\left(1.96\right)}^{\text{2}}\times 0.37\times 0.63/{\left(0.05\right)}^{\text{2}}\\ & \text{= }358 \end{array}$$

Where,

n = Sample size
Z = 1.96 at 95% Confidence Interval
p = past prevalence, 37%^2^
e = margin of error, 5%

The convenient sampling method was used. The data was collected by house-to-house visits using structured questionnaires. Economic status was assessed by using Kuppuswamy's socioeconomic scale^4^ using three variables education, occupation, and family income per month. The health statuses of respondents were recorded by history taking and from the prescription of medicine taken. In psychological problems, to assess depression status, Beck Depression Inventory (BDI)^5^ was used. The collected data were analyzed by using Statistical Package for the Social Science (SPSS) version 20.

## RESULTS

The prevalence of early marriage was 187 (52.2%) (47.03 to 57.37 at 95% confidence interval). Health consequences were seen in 256 (71.5%) participants. Among the health problem, 116 (62%) of early marriage women had gynaecological problem followed by depression problem 85 (45.5%) miscarriage 32 (17.1%). Women who got early marriage had 3 times more chances of having gynaecological, miscarries/ stillbirth, and depression problems (Table 1).

**Table 1:** Health problems of respondents (n = 187).

Health problems	Early marriage n (%)
Gynecological	116 (62.0)
Miscarries/ Still birth	32 (17.1)
Hemorrhage	23 (12.3)
Depression	85 (45.5)

Most of the respondents 150 (41.9%), were from the age group 20-29 years followed by 30-39 years 126 (35.7%) and 40-49 years 53 (14.8%). The mean age of the respondent was 25.7 years with 14.1 years of standard deviation. The prevalence of early marriage was 187 (52.2%). The mean age of marriage was 17.2 years (Table 2).

**Table 2:** Age wise distribution by respondents.

Age group (in years)	Early marriage n (%)
Less than 20	15 (4.2)
20-29	77 (21.5)
30-39	67 (18.7)
40-49	28 (7.8)
Total	187 (52.2)

The majority 167 (89.3) of respondents who married earlier were Hindu by religion. Women from the Hindu religion have 2.5 times more chances of early marriage compared to other religions. The present study revealed that 218 (60.1%) of women were literate. Early marriage was observed in 104 (55.6%) among illiterate women and there were 4.69 times more chances of early marriage in illiterate women. The education status of women is a significant factor in early marriage. Occupational distribution found that majority of mothers were farmer 198 (55.3%) followed by business 76 (21.2%) and house makers 56 (15.6%). In occupation, women from farmer families have slightly higher chances of early marriage with compared to other occupations but not a significant difference. More than half of respondents 199 (55.6%) belonged to a low level of economic status (Table 3).

**Table 3:** Demographic profile of respondents.

Variables		Early Marriage n (%)
Religion	Hindu	167 (89.3)
	Others	20 (10.7)
Education status	Illiterate	104 (55.6)
	Literate	83 (44.4)
	Farmer	115 (61.5)
	Business	32 (17.1)
Occupation	Service	6 (3.2)
	Students	4 (2.2)
	Housemaker	30 (16.0)
Economic status	Low	130 (69.5)
	Lower-middle	57 (30.5)

## DISCUSSION

Early marriage is recognized globally as a public health problem. Women in Nepal marry at an earlier age than men and the median age at first marriage for women is 17.9 years^2^ which is consistent with the result of the present study where the median age of marriage was 17.2 years. The mean age at first marriage of the present study was found higher than the mean age of marriage of women in Bangladesh where it was 15.7 years. This difference may be due to a different time and a high prevalence of child marriage in Bangladesh. A study conducted by Shah R et al. at Dhankuta Municipality found that more than half of women were married before the legal age of 18 years.^6^ A similar result was found in the present study that the prevalence of early marriage was 52.2%. It is higher than the prevalence of child marriage was 20.7% in Ghana.^7^

In the present study, the majority 167 (89.3%) of respondents who married earlier were Hindu by religion. Women from the Hindu religion have 2.5 times more chances of early marriage compared to other religions and it was found a significant difference with a 95% confidence interval of 1.42 - 4.56. This finding is consistent with the finding of Paul P where it was 82.7%.^8^

A present study revealed the 60.1% of women were literate which is consistent with the finding of Paul P (61.5%)^8^ and lower than the result of NDHS where 69% of women were literate.^2^ Education is a key factor for delaying sexual activity, pregnancy, marriage, and childbearing. In the present study, education was found a significant factor of early marriage which is consistent with the finding of Sah and Kamal.^6,9^ Keeping girls in school or vocational training not only helps protect them from pregnancy, illness, and death but also enhances their earning potential and socioeconomic status. Educated girls can contribute to the health and welfare of their family and marry men of their choosing and age.^10^

A study conducted by Roy in Bangladesh also found similar that “girls from the poorest family were almost twice as likely to be married before age 18 than girls from the richest family.^11^ A study conducted by Sah RB et al. at Dhankuta Municipality found that economic status was found significant with early marriage.^6^ In South Asia, poverty plays a crucial role in perpetuating early marriage. Daughters are considered an economic burden for the family. Feeding, clothing, and educating girls is costly, and daughters will eventually leave the parents' house. Births from early married women are said to be "too soon, too close, and too many".

In the present study, miscarriage was found in 17.1% of early married women, which is consistent with the finding of Paul, that is, 19.5%.^8^ Sixty-two percentage of early marriage women had gynecological problems and 45.5% had depression problems. Gynaecological and depression problems were found a significant difference with early marriage and three times more in early marriage women. Hemorrhage was no significant difference with early marriage. Early marriage plays an important role in a deadly disease, cervical cancer. Common risks for cervical cancer are early marriage, low socioeconomic status, poor access to health care, poor genital hygiene, and husbands who had multiple sex partners.^3^

## CONCLUSIONS

The present study revealed that the prevalence of early marriage was high. It is observed that early married women had a lower level of socio-economic status, lower level of education, which harmed their health. Early married women had gynecological, miscarries/ stillbirth, and depression as common health problems. Early married women have lower participation in education, fewer opportunities for employment and training. To end the early marriage, it requires a multifactor approach focused on the girls, their families, religion, community leaders, and the government. The programs should provide families and communities with education and reproductive health services that can help to reduce early marriage, early pregnancies, and illness and death in young mothers and their children. To break the cycle of poverty, programs are needed to educate and empower women. In addition to poorer and less educated women must be recognized in policy and program as they are more vulnerable to adverse reproductive health risks.
